# Identification of pesticide varieties by testing microalgae using Visible/Near Infrared Hyperspectral Imaging technology

**DOI:** 10.1038/srep24221

**Published:** 2016-04-13

**Authors:** Yongni Shao, Linjun Jiang, Hong Zhou, Jian Pan, Yong He

**Affiliations:** 1 College of Biosystems Engineering and Food Science, Zhejiang University, Hangzhou 310058, China

## Abstract

In our study, the feasibility of using visible/near infrared hyperspectral imaging technology to detect the changes of the internal components of *Chlorella pyrenoidosa* so as to determine the varieties of pesticides (such as butachlor, atrazine and glyphosate) at three concentrations (0.6 mg/L, 3 mg/L, 15 mg/L) was investigated. Three models (partial least squares discriminant analysis combined with full wavelengths, FW-PLSDA; partial least squares discriminant analysis combined with competitive adaptive reweighted sampling algorithm, CARS-PLSDA; linear discrimination analysis combined with regression coefficients, RC-LDA) were built by the hyperspectral data of *Chlorella pyrenoidosa* to find which model can produce the most optimal result. The RC-LDA model, which achieved an average correct classification rate of 97.0% was more superior than FW-PLSDA (72.2%) and CARS-PLSDA (84.0%), and it proved that visible/near infrared hyperspectral imaging could be a rapid and reliable technique to identify pesticide varieties. It also proved that microalgae can be a very promising medium to indicate characteristics of pesticides.

Pesticides, which are widely used in agriculture to prevent, eliminate and control damages caused by pests or weeds, play an important role in enhancing the productivity of agricultural products^1^. Pesticides can benefit the development of agricultural production, but on the other hand, they also bring much negative influence on environment and human health^2^. Pesticides can pollute soil, water, and vegetation, and they are harmful to organisms like fish, beneficial insects and plants^3^. Furthermore, human health would suffer a huge threat from the water which was polluted by pesticides. As a result, it becomes more and more essential to identify and manage pesticides properly in water to keep a sustainable ecosystem for human beings and other lives.

Identifying the varieties of pesticides is the first challenge during the pesticide management. It will help to find the cause of pollutions and a solution to tackle pollution issues. As far as we know, chromatography is the most popular method to detect pesticide varieties. It is also the most accurate and sensitive method for water contamination detection. But the disadvantages of this approach are that it always involves complex sample preparations and high cost. Besides chromatography, there are other methods that can detect pesticides, such as immunoassay^4^ and fluorescence spectrometry^5^. However, those methods require a very sensitive detector to secure the accuracy of detection, and volatile or decomposable pesticides are difficult to be detected by some of these technologies. In addition, these technologies are inappropriate when continuous monitoring of water pollution is required. So it becomes more and more urgent and necessary to find out a rapid and reliable but cost-effective method to monitor the water pollution and identify pesticides varieties for pest management.

Microalgae are the most common microorganisms in the equilibrium of aquatic ecosystems, and play a significant role in the first level of the food chain in rivers or lakes. As most algae are light autotrophic organisms, photosynthesis is an important procedure in cell metabolism, but the accumulation of pigments during photosynthesis will be affected by the toxins of pesticides. Meanwhile, the generation of other components of algae could also be affected when photosynthesis is not proceeding properly. Algae are often used in tracing and detecting harmful substances in water as they can provide the information of pollutants in water organisms which are sensitive to the toxicants^6^,^7^,^8^, and low detection limits can be reached^9^. The information of pollution levels can be also reflected by microalgae in biosensors according to modifications in metabolic or photosynthetic activities^10^. In our study, the change of components (mainly pigments) of *Chlorella pyrenoidosa* was used as an indicator to detect pesticides varieties, and it has high stability in producing biological signals^9^.

As a combined technology of sensing, computing and information processing, hyperspectral imaging technology is becoming more and more popular in applications because of its attractive features like nondestructive detection and high efficiency. It can achieve the covering of continuous spectrum by imaging and spectroscopic detection. The spectra acquired from samples can provide much complex structural information which is related to the vibration behavior of bonds, like the molecular bonds C-H, N-H and O-H^11^. Due to these benefits and its intrinsic characteristics, hyperspectral imaging technology has been widely used in a wide array of applications. It was recently applied to detect agricultural products, such as rice^12^, meats^13^ and oils^14^. Besides those mentioned applications, hyperspectral imaging technology also was used in the field of remote sensing, and it has been successfully applied to estimating water quality in lakes and reservoirs by detecting chlorophyll-a of microalgae^15^,^16^,^17^. The intracellular distribution of pigments in *H. lacustris* (*Chlorophyceae*) were obtained by the hyperspectral imaging together with microscope^18^. In our study, the visible/near infrared (Vis/NIR) hyperspectral imaging technology was used to detect the characteristics of *Chlorella pyrenoidosa* so as to identify the pesticide varieties.

The objective of this study was to quest the potentiality of using Vis/NIR hyperspectral imaging technology to instantly detect the components in freshly harvested *Chlorella phyrenoidosa* and then to identify the varieties of pesticides. The emphasis of the study was on (1) detecting the spectral variation of four different prepared samples corresponding to three pesticides (glyphosate, butachlor, atrazine) plus a normal medium, (2) extracting the hyperspectral data from interesting regions of samples, and choosing the useful wavelengths for pesticide varieties identification, (3) comparing the prediction accuracy of different modeling approaches i.e. partial least squares discriminant analysis combined with full wavelengths (FW-PLSDA) model, partial least squares discriminant analysis combined with competitive adaptive reweighted sampling algorithm (CARS-PLSDA) model and linear discrimination analysis combined with regression coefficients (RC-LDA) model, and proposing the best model to identify pesticide varieties.

## Results

### The detection of Chlorophyll a of samples

**Figure 1:**
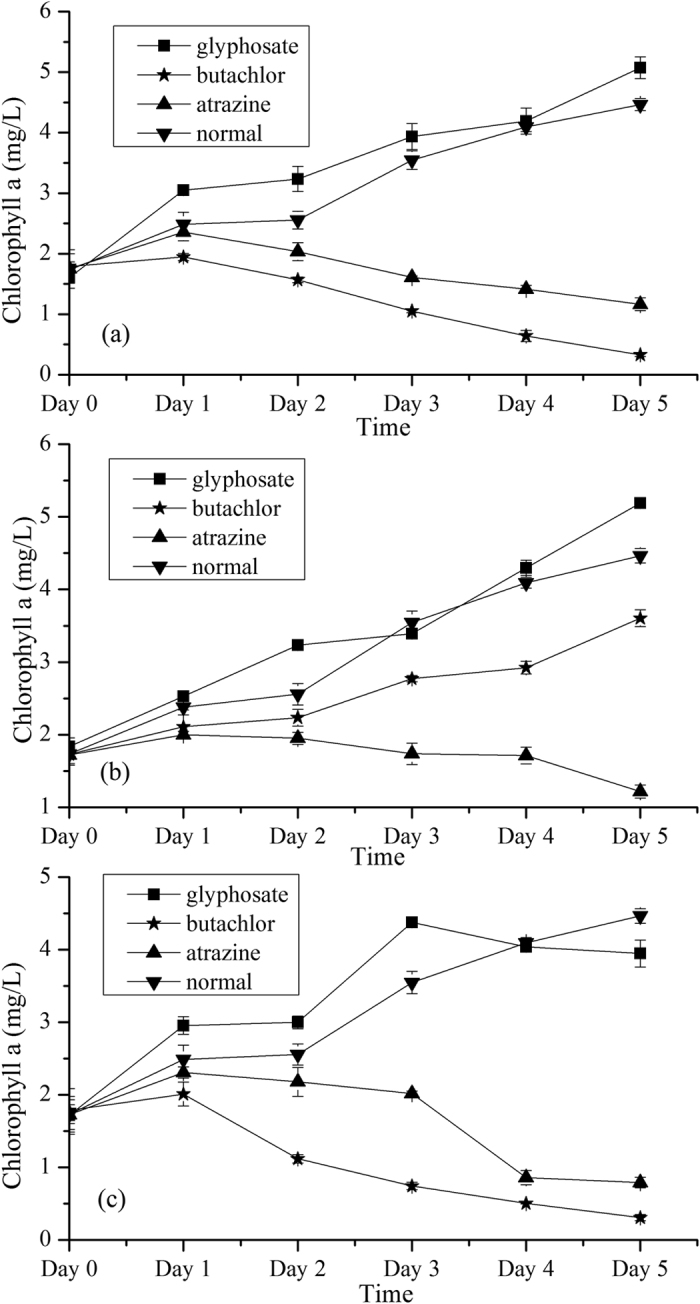
Dynamic changes of chlorophyll a of *Chlorella pyrenoidosa* cultured in media with pesticides (butachlor, atrazine and glyphosate) of three concentrations (**a–c**) and water from day 0 to day 5. (**a**) 0.6 mg/L; (**b**) 3 mg/L; (**c**) 15 mg/L.

Chlorophyll a is the main component in *Chlorella pyrenoidosa* cells, and changes of chlorophyll content can indirectly represent the change of internal elements in algae which may be affected by pesticides. The dynamic changes of chlorophyll a of *Chlorella pyrenoidosa* cultured in media with three concentrations of pesticides (butachlor, atrazine and glyphosate) and normal water during six days are shown in Fig. 1. Low concentrations of glyphosate promoted the algae growth as shown in Fig. 1(a,b). The chlorophyll content in algae, when exposed to the high concentration (15 mg/L) of glyphosate for a long time, decreased after day 3 as show in Fig. 1(c). For pesticide butachlor, although the concentration of 0.6 mg/L promoted the algae growth from day 0 to day 5, it impeded the growth of algae as compared with the normal water medium. The chlorophyll content in algae, when exposed to higher concentrations (3 mg/L, 15 mg/L) of butachlor decreased after day 1 as shown in Fig. 1(b,c). *Chlorella pyrenoidosa* seemed to be more sensitive to atrazine, and all three concentrations caused the decrease of chlorophyll accumulation after day 1. The responses from microalgae cultured in different types and concentrations of pesticides were different to each other by the comparison of chlorophyll a. As one part of internal components in microalgae, the information of chlorophyll a would be useful for identifying the varieties of pesticides.

### Spectral features of samples

**Figure 2:**
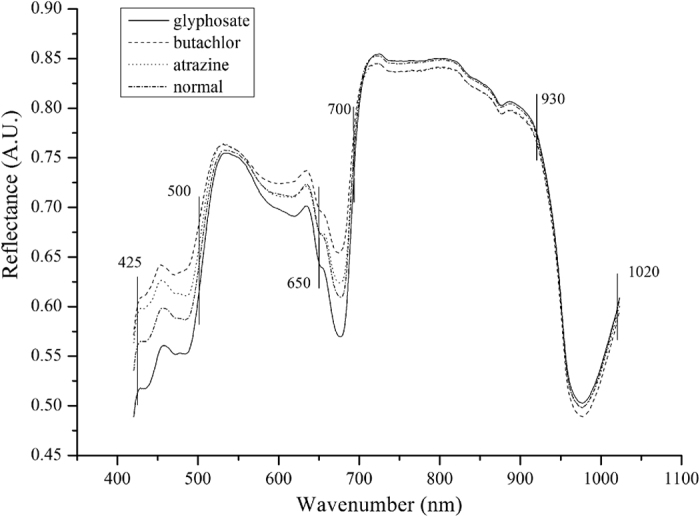
The average visible and near infrared spectra of four samples in 3 mg/L on day 1.

The spectra in the Vis/NIR region contain much rich but complex information of samples related to certain vibration behavior of molecular bonds (e.g. C–H, O–H). These characteristics can be explored to predict samples polluted by different pesticides. The mean spectra (420–1020 nm, the wavelengths of 380–420 nm were excluded for noisy signals) of samples cultured in water and 3 mg/L of three types of pesticides on day 1 are shown in Fig. 2. A significant absorption band was observed between 930 and 1020 nm related to O–H third stretching overtone^19^. Another obvious low reflectance between 650 nm and 700 nm might be related to the algal chlorophyll^15^. The front part of spectra between 425 nm and 500 nm were associated with some pigments, such as carotenoid and chlorophyll^20^. Combining Figs 1 and 2, wavelengths between 650 nm and 700 nm reflected the content of chlorophyll a. The figures showed that the content of chlorophyll a in microalgae polluted by glyphosate was higher than those by atrazine and butachlor, and the reflectance of glyphosate was lower than other pesticides. The hyperspectral reflectance characteristic of microalgae had also proved strong correlations with the concentrations of microalgae chlorophyll^21^,^22^,^23^.

For all samples, the spectral profiles of *Chlorella pyrenoidosa* cultivated in four different media had similar patterns, and no prominent peak value was found. However, the spectral curves acquired from different samples might be overlapped which will make it much more difficult to identify pesticides by observed spectral data directly. To solve the problem, it became necessary to eliminate the useless and overlapped spectral data by chemometric.

### Spectral analysis by PCA

**Figure 3:**
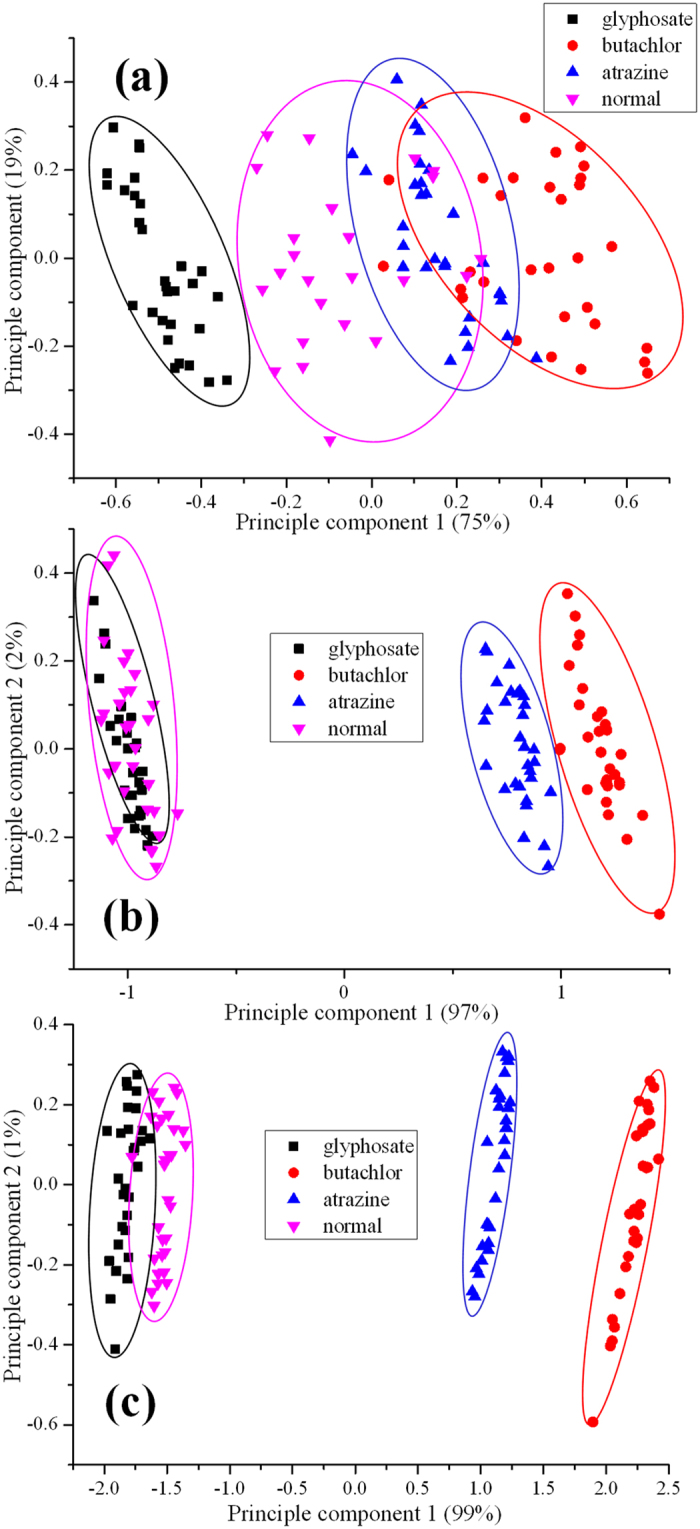
The scores scatter plots of PC1 and PC2 from the spectrum obtained from microalgae cultured in media with pesticides at the concentration of 3 mg/L and water for three periods respectively. (**a**) day 1; (**b**) day 3; (**c**) day 5.

Principle component analysis (PCA) is a technique to simplify data by extracting the most important element and effective structure^24^. It has been widely used to process hyperspectral data^13^,^25^. In our study, PCA was applied to acquire any variation among samples attributed to difference in their spectral data. The first two principle components (PCs) explained up to 94%, 99% and 100% of the variations among the spectral data for the different samples on day 1, 3 and 5 as shown in Fig. 3. There was a significant overlapping among those four different samples on the same day. Specifically, the glyphosate and normal water were distributed almost at the same location on day 3 as shown in Fig. 3(b). So, it became critical and challenging to build the optimal model to identify pesticide varieties faced with redundant information.

### Identification model based on FW-PLSDA

In the study, partial least squares discriminant analysis combined with full wavelengths (420–1020 nm, 478 variables) (FW-PLSDA, introduced in section Experimental Procedures) model was performed to identify the pesticide varieties. In order to build the identification model, the calibration and prediction set were prepared. Taking the data on day 1 as an example, we randomly selected 80 samples from four different media (20 samples for each medium) as the calibration set, and the rest 40 samples (10 samples for each medium) were used as the prediction set. The same division of calibration and predication was also applied to the data acquired on day 2 to day 5. In addition, both the CARS-PLSDA and RC-LDA models established in the following sections use this approach to select the calibration and prediction sets.

The identification results for the four media are shown in Table 1. The correct classification rates (CCRS) of the first three days were low, and they were 61.7%, 63.3% and 65.0%. The reasons might be that the internal component change in *Chlorella pyrenoidosa* polluted by different pesticides was not significant, or some spectral data showed some overlapping information^26^. Although the average CCRS values of day 4 and day 5 seemed to be better and reached 87.5% and 83.3% respectively, the average CCR only reached 72.2%. For further analysis, competitive adaptive reweighted sampling (CARS) algorithm was applied to eliminate the influence of the less important variables or overlapped information, and CARS-PLSDA was used to build the model to identify pesticide varieties.

### Identification model based on CARS-PLSDA

**Figure 4:**
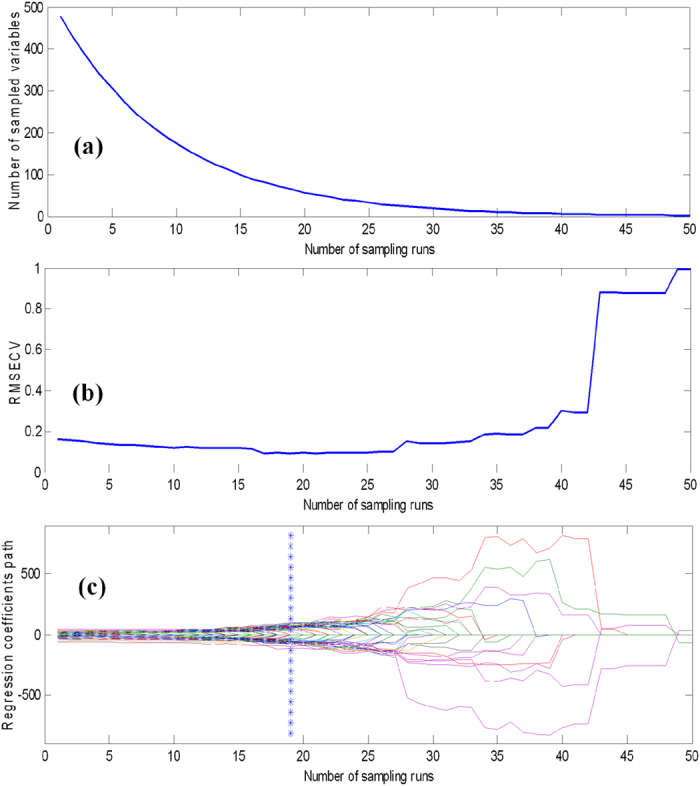
The processing of effective variables selection by the CARS method.

During the experiment, competitive adaptive reweighted sampling (CARS) algorithm was introduced to minimize the dimensionality of hyperspectral data and select the effective variables (wavelengths). Data at the concentration of 0.6 mg/L on day 2 were chosen to select effective variables, and the process of selecting effective variables using CARS was shown in Fig. 4. In the study, the sampling was done by Monte Carlo (MC) method and the iteration number was set to 50. Figure 4(a) shows the tendency number for sample variables. Due to the influence of exponentially decreasing function (EDP), the number of sample variables decreased rapidly during the initial 30 sampling runs, but showed smooth and slow change after 30 sampling runs. This indicated that CARS had two steps, which include 1) selecting variables roughly and 2) selecting variables precisely. In Fig. 4(b), the 10-RMSECV values firstly showed a slightly downward trend because of the elimination of the variables which have less information, and the RMSECV value was the lowest in the 19^th^ sampling run. Due to the excluding of some effective variables, the RMSECV values increased obviously after the 19^th^ sampling run. Figure 4(c) showed the regression coefficients (RC) path during the sampling by the MC method, and the 478 variables selected in the sampling process were represented by different colored lines. The number of sampling runs where the line marked by asterisk (in the 19^th^ sampling run) corresponded to the lowest RMSECV value and high RC value. When the number of sampling runs increased, the RC values for several variables dropped slightly to zero because they are carrying no useful information for building model, and wiped out from the selection of effective variables.

During the procedure of running sampling, the subset of effective variables was determined based on the lowest RMSECV value which was found in the 19^th^ sampling run, and the optimized subset including 71 variables was used to build the CARS-PLSDA model. The approach to build the calibration and prediction sets was the same as was done with the FW-PLSDA model, and the result of the CARS-PLSDA model is shown in Table 2. The values of CCRS for day 1 to day 5 were higher than the ones achieved by the FW-PLSDA model, while the average CCRS value of 80.0% on day 2 and 74.2% on day 3 were lower. However, the average CCR of five days was improved to 84% which was higher than the result using the FW-PLSDA model. It indicated that changes of the internal components (mainly pigments) of *Chlorella pyrenoidosa* polluted by different pesticides could be identified by this approach. Furthermore, selecting effective variables (wavelengths) was an essential procedure to build a more accurate and stable model for pesticide varieties identification.

### Identification model based on the RC-LDA model

**Figure 5:**
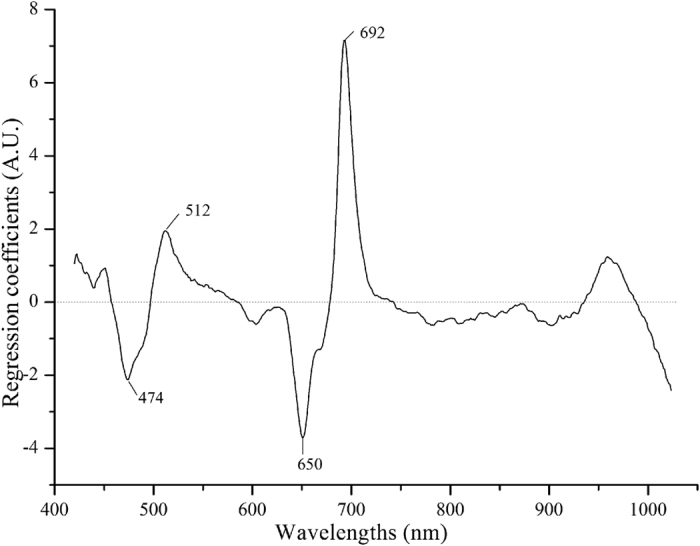
The effective wavelengths selected by regression coefficients.

Although the identification model built by CARS-PLSDA seems better than other models, the disadvantage of requiring 71 variables (wavelengths) to establish a model is still unacceptable in some cases for taking much more calculation time, and on the other hand, its identification accuracy was not high enough. Therefore, an optimal model which consumes less time and has higher identification accuracy seems much more attractive and necessary. The regression coefficient of the variables (wavelengths) plays a rather important role in the PLS regression^27^. The absolute values of the peaks indicate the contribution of wavelengths at these positions in regression model. Wavelengths with high regression coefficient were selected for further analysis, but wavelengths with small coefficients were excluded for little contribution to improving the productivity of the model. In the study, the calibration model was built using all hyperspectral preprocessing data at a concentration of 15 mg/L on day 5, and four effective variables (474, 512, 650 and 692 nm) were selected by regression coefficients which is shown in Fig. 5. Compared to the wavelengths selected by CARS, the number of variables selected by regression coefficients was obvious less and more redundant information was excluded. Furthermore, more useful information was also picked out. In Fig. 5, the wavelength at 474 nm was associated with carotenoids b^28^, and 512, 650 and 692 nm might be related to chlorophyll^19^,^29^,^30^. LDA combined with effective variables selected by regression coefficients was applied in our investigation to build another model to identify the pesticides.

The result achieved by identification model of RC-LDA is shown in Table 3. The highest CCRs for those three days can reach up to 100%, and the lowest rate can also reach to 91.7%, which was much higher than those of the FW-PLSDA or CARS-PLSDA model. The average CCR for the four different media was 97.0%. Therefore, the effective wavelengths extract by RC had great contribution on identifying the pesticide varieties.

In the study, the classification accuracy and stability were obvious disparity among the FW-PLSDA, CARS-PLSDA and RC-LDA models and the performances of the three models at three concentrations were compared among the three tables. The CARS-PLSDA model seemed to be more reliable than FW-PLSDA for its average CCRS of different concentrations from day 1 to day 5. Although the RC-LDA model only combined with four effective variables which was much less than 478 variables of the FW-PLSDA model and 71 variables of the CARS-PLSDA model, it showed a strong stability and reliability with higher average CCRS for identifying pesticide varieties. The results proved that using hyperspectral imaging technology to identify the pesticide varieties is feasible and the RC-LDA model was the optimal classification model.

## Discussion

Pesticide’s impact on microalgae is complex as pesticides not only hinder the growth of biomass, but also damage the accumulation of internal components. The most obvious components are pigments such as chlorophyll which is essential to microalgae photosynthesis. It is useful to identify varieties of pesticides by using the hyperspectral imaging technology to acquire the information of these components. By acquiring the hyperspectral imaging of microalgae, which are polluted by pesticides (such as butachlor, atrazine and glyphosate), the micro-structure of microalgae can be investigated to find out the influence from pesticides and eventually facilitate the detection of pesticide varieties. Meanwhile, *Chlorella pyrenoidosa* is an excellent medium which carries lots of interesting information, and investigation on *Chlorella pyrenoidosa* can give good indication on the water quality level.

The study above was performed in the lab where the environment is controllable and favorable for pesticide variety identification. But in reality like in the field, microalgae may be affected by other polluted substances, including heavy metals^31^,^32^,^33^ and eutrophic substances^34^,^35^. In such cases, technology like chromatography to recognize different components may be needed in advance. So far, there has been no report using hyperspectral imaging technology to identify pesticide varieties by detecting the internal components (mainly pigments) of microalgae. Our study showed that hyperspectral imaging technology is promising to identify pesticide varieties, and it is an accurate and efficient approach for long-term monitoring of water pollution due to pesticides. But as mentioned in previous sections, there were still some constraints of our study. Moving forward, we would focus on the limitation of this technology and explore it with other technologies to detect pesticide in polluted water in applications where the environment is uncontrollable and much more complicated.

## Methods

### Algae cultivation and sample preparation

*Chlorella pyrenoidosa* (FACHB-11) was purchased from the freshwater algae culture collection at the Institute of Hydrobiology of China. Algae were cultivated in BG11 medium in 2 L Erlenmeyer flasks. Three pesticides were chosen in this study and they were glyphosate (30% glyphosate content, Jingma Chemicals Co., Ltd.), butachlor (60% active ingredient, Sino pesticide Co., Ltd.) and atrazine (90% active ingredient, Jinan Lvba pesticides Co., Ltd.). All three pesticides are translocated herbicides.

Four different media were set, three of which contained pesticides (glyphosate, butachlor and atrazine) and the other one was normal medium as the control. The same concentration of *Chlorella pyrenoidosa* was cultivated in the four media. The pesticide concentrations were set to be 0.6 mg/L, 3 mg/L and 15 mg/L respectively. The beginning algal concentration was about 7.08 × 10^8^ cells/mL which was determined by cell counting. All samples were set to the artificial climate chamber where temperature was set at 25 ˚C and the illumination level was between 2500 and 3500 lx.

Samples were examined in day 1 to day 5 where 5 mL of each medium was added to the same size of glass dishes (d = 33 mm). Images of different samples were acquired individually by the visible/near infrared hyperspectral imaging system. During the same day, there were 30 samples for each medium and 300 samples were acquired in all.

### Hyperspectral imaging system

The visible/near infrared hyperspectral imaging system in reflectance mode was employed to obtain hyperspectral images of samples. The system consists of an imaging spectrograph (ImSpectorV10E, Spectral Imaging Ltd., Finland) which can acquire images with wavelength range from 380 to 1020 nm, a high performance CCD camera, a conveyer platform used for carrying and moving samples and a computer supported with Spectral-Cube data acquisition software (Spectral Imaging Ltd., Finland) to control the stepper motor speed, exposure time, binning mode and image acquisition. An illumination unit containing two line illuminations (SchottFostec-A0891, SchotteFostec Ltd., USA) was installed above the conveyer platform. The camera has 672 × 512 (spatial × spectral) pixels with a spectral resolution of 2.8 nm.

### Imaging acquisition and correction

The exposure time was 0.09 s and the distance between the lens of CCD camera and the conveyor which was used to carry samples was 310 mm. Each glass dish was placed on the conveyor stage and moved at a speed of 3.1 mm/s to be scanned line by line using the visible/near infrared hyperspectral imaging system to build a hyperspectral image (I_0_) with dimension (*x*, *y*, *λ*). There into, *x* is the number of rows in pixels of spatial dimensions, *y* is the number of columns in pixels of spatial dimensions and *λ* is the number of wavebands. In the experiment, images were obtained with 672 pixels in *x*-direction, 512 wavelengths in *λ*-direction at 1.2 nm intervals between continuous bands. To reduce the influence of the dark current of the camera and environment of instrument, the raw hyperspectral images were corrected with white and black reference images as the following equation:


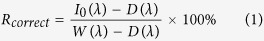


where *R*_*correct*_, *I*_*o*_ are the corrected and the raw hyperspectral image respectively, and *λ* is the number of wavelength. *W(λ)* is the white reference image obtained from a white Teflon board (CAL-tile 200, 200 mm × 25 mm × 10 mm) with the reflectance factor of about 99%. *D(λ)* is the dark reference image with the reflectance factor of 0% obtained by turning off the light and covering the camera lens with its lid completely.

### Spectral data extraction and processing

The calibrated hyperspectral images were then imported to the Environment for Visualizing Images (ENVI) software (ITT Visual Information Solutions, Boulder, USA) for image analysis. The size 135 × 150 pixel at the center of an image was chosen by the region of interest (ROI) tool. The reflectance spectra curve of pixels extracted from ROI regions were averaged to represent each sample.

For the purpose of eliminating noise of the spectral data and improve the predictive ability of samples, the preprocessing methods of Savitzky-Golay smoothing was used. The spectral data were calculated and processed by Unscrambler X 10.1 (CAMO Software, Norway). The preprocessed spectral data were used to establish FW-PLSDA, CARS-PLSDA and RC-LDA respectively.

### Determination of chlorophyll a

Chlorophyll a of *Chlorella pyrenoidosa* was measured by the ultrasound-assisted hot-ethanolextraction method^36^. The chlorophyll a was estimated using the equations as the following:


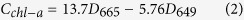


where *C*_*chl-a*_ was the concentration of chlorophyll a as mg/L in the original sample, and *D* was the absorbance.

### Partial least-squares discrimination analysis (PLSDA)

Partial least-squares (PLS) analysis has been developed to be a standard tool in chemometrics and is used widely in Vis/NIR spectral analysis^37^,^38^,^39^. In the study, the model that predicts the class number for each sample established by partial least-squares discrimination analysis (PLSDA) could be applied to identify different classes based on the PLS method. The spectra after preprocessing by SG smoothing were treated as matrix *X*. Four kinds of variables were set as matrix *Y* (glyphosate medium-1, butachlor medium-2, atrazine medium-3, normal medium-4). The threshold, which is usually set at 0.5^40^, was set as ±0.3 for recognition, which means the predicted value minus the measured value (*Y* value) should be in the range of −0.3 to 0.3. Several parameters were used to estimate the predictive capabilities and accuracy of the models, such as the correlation coefficient (r), the root-mean-square error of calibration (RMSEC), and the root-mean-square error estimated by cross-validation (RMSECV). An optimal model should have a high value of r, and low RMSEC and RMSECV.

### Competitive adaptive reweighted sampling

Competitive adaptive reweighted sampling (CARS) algorithm is a useful method to select informative variable from the full spectrum combined with partial least squares (PLS) regression. It has been used widely to deal with hyperspectral data. The variables were selected from the large absolute values of regression coefficients in the PLS model by the CARS algorithm which combined exponentially decreasing function (EDF) and adaptive reweighted sampling (ARS). The variables were removed for their low weights and the subset with the lowest root mean square error of cross-validation (RMSECV) was considered as the best variable subset after the cross-validation. Meanwhile, Monte Carlo method applied to numerical calculation widely was used to the sampling of the hyperspectral data in the study. Details of the CARS methodology can be found in the literature^41^. The CARS algorithm was used to extract key wavelengths from the Vis/NIR spectrum in this study.

### Linear discriminant analysis

Linear discriminant analysis (LDA) is a statistical method for feature extraction and classification^42^. It has been successfully applied in pattern recognition, machine learning, computer vision^43^, and hyperspectral image^44^,^45^. In order to achieve the classification for information and extract the compressed space dimension, LDA is used to project the high-dimensional pattern onto the vector space which has the best discrimination effect. The scatter matrix between different classes is the biggest and within same class is the smallest after projecting by LDA. In this study, the LDA model was established by the selected wavelengths which had high regression coefficients.

## Additional Information

**How to cite this article**: Shao, Y. *et al*. Identification of pesticide varieties by testing microalgae using Visible/Near Infrared Hyperspectral Imaging technology. *Sci. Rep.* **6**, 24221; doi: 10.1038/srep24221 (2016).

## Figures and Tables

**Table 1:** Pesticide varieties identification results for day 1 to day 5 by the FW-PLSDA model.

Time	Concentrations (mg/L)	Latent variables (LVs)	Calibration set (80)		Prediction set (40)		
			r_c_	RMSEC	r_p_	RMSEP	CCR
Day 1	0.6	10	0.994	0.123	0.992	0.179	87.5%
	3	10	0.994	0.130	0.985	0.374	32.5%
	15	9	0.967	0.283	0.963	0.315	65.0%
	average						61.7%
Day 2	0.6	7	0.982	0.213	0.966	0.290	65.0%
	3	7	0.986	0.185	0.966	0.360	60.0%
	15	11	0.991	0.148	0.981	0.279	65.0%
	average						63.3%
Day 3	0.6	9	0.985	0.195	0.971	0.295	67.5%
	3	9	0.990	0.160	0.972	0.368	60.0%
	15	7	0.986	0.188	0.966	0.292	67.5%
	average						65.0%
Day 4	0.6	10	0.989	0.163	0.982	0.215	82.5%
	3	6	0.987	0.180	0.988	0.178	90.0%
	15	8	0.991	0.150	0.990	0.165	90.0%
	average						87.5%
Day 5	0.6	5	0.987	0.181	0.978	0.231	77.5%
	3	8	0.988	0.173	0.981	0.219	80.0%
	15	5	0.991	0.150	0.992	0.983	92.5%
	average						83.3%
Average of 5 days							72.2%

**Table 2:** Pesticide varieties identification results for day 1 to day 5 by the CARS-PLSDA model.

Time	Concentrations (mg/L)	Latent variables (LVs)	Calibration set (80)		Prediction set (40)		
			r_c_	RMSEC	r_p_	RMSEP	CCR
Day 1	0.6	10	0.992	0.140	0.988	0.174	92.5%
	3	9	0.989	0.167	0.983	0.215	85.0%
	15	11	0.986	0.189	0.849	0.708	85.0%
	average						87.5%
Day 2	0.6	9	0.991	0.150	0.987	0.183	85.0%
	3	7	0.984	0.202	0.965	0.298	70.0%
	15	10	0.983	0.203	0.979	0.230	85.0%
	average						80.0%
Day 3	0.6	7	0.984	0.201	0.977	0.251	77.5%
	3	9	0.990	0.159	0.981	0.317	70.0%
	15	7	0.987	0.181	0.976	0.245	75.0%
	average						74.2%
Day 4	0.6	10	0.993	0.134	0.990	0.161	95.0%
	3	5	0.988	0.172	0.984	0.200	85.0%
	15	9	0.991	0.147	0.986	0.187	92.5%
	average						90.8%
Day 5	0.6	5	0.989	0.161	0.984	0.200	90.0%
	3	8	0.988	0.172	0.980	0.223	85.0%
	15	5	0.986	0.187	0.987	0.181	87.5%
	average						87.5%
Average of five days							84.0%

**Table 3:** Pesticide varieties identification results for day 1 to day 5 by the RC-LDA model.

Time	Concentrations (mg/L)	Calibration			Prediction		
		No.	Missed	CCR	No.	Missed	CCR
Day 1	0.6	80	0	100.0%	40	1	97.5%
	3	80	0	100.0%	40	4	90.0%
	15	80	0	100.0%	40	1	97.5%
	average						95.0%
Day 2	0.6	80	0	100.0%	40	0	100.0%
	3	80	2	97.5%	40	8	80.0%
	15	80	2	97.5%	40	2	95.0%
	average						91.7%
Day 3	0.6	80	0	100.0%	40	1	97.5%
	3	80	0	100.0%	40	1	97.5%
	15	80	0	100.0%	40	0	100.0%
	average						98.3%
Day 4	0.6	80	0	100.0%	40	0	100.0%
	3	80	0	100.0%	40	0	100.0%
	15	80	0	100.0%	40	0	100.0%
	average						100.0%
Day 5	0.6	80	0	100.0%	40	0	100.0%
	3	80	0	100.0%	40	0	100.0%
	15	80	0	100.0%	40	0	100.0%
	average						100.0%
Average of five days							97.0%
